# *Msh2* Acts in Medium-Spiny Striatal Neurons as an Enhancer of CAG Instability and Mutant Huntingtin Phenotypes in Huntington’s Disease Knock-In Mice

**DOI:** 10.1371/journal.pone.0044273

**Published:** 2012-09-07

**Authors:** Marina Kovalenko, Ella Dragileva, Jason St. Claire, Tammy Gillis, Jolene R. Guide, Jaclyn New, Hualing Dong, Raju Kucherlapati, Melanie H. Kucherlapati, Michelle E. Ehrlich, Jong-Min Lee, Vanessa C. Wheeler

**Affiliations:** 1 Center for Human Genetic Research, Massachusetts General Hospital, Boston, Massachusetts, United States of America; 2 Department of Medicine, Brigham and Women’s Hospital, Boston, Massachusetts, United States of America; 3 Farber Institute for Neurosciences, Thomas Jefferson University College of Medicine, Philadelphia, Pennsylvania, United States of America; Ohio State University, United States of America

## Abstract

The CAG trinucleotide repeat mutation in the Huntington’s disease gene (*HTT*) exhibits age-dependent tissue-specific expansion that correlates with disease onset in patients, implicating somatic expansion as a disease modifier and potential therapeutic target. Somatic *HTT* CAG expansion is critically dependent on proteins in the mismatch repair (MMR) pathway. To gain further insight into mechanisms of somatic expansion and the relationship of somatic expansion to the disease process in selectively vulnerable MSNs we have crossed *HTT* CAG knock-in mice (*HdhQ111*) with mice carrying a conditional (*floxed*) *Msh2* allele and *D9-Cre* transgenic mice, in which Cre recombinase is expressed specifically in MSNs within the striatum. Deletion of *Msh2* in MSNs eliminated Msh2 protein in those neurons. We demonstrate that MSN-specific deletion of *Msh2* was sufficient to eliminate the vast majority of striatal *HTT* CAG expansions in *HdhQ111* mice. Furthermore, MSN-specific deletion of *Msh2* modified two mutant huntingtin phenotypes: the early nuclear localization of diffusely immunostaining mutant huntingtin was slowed; and the later development of intranuclear huntingtin inclusions was dramatically inhibited. Therefore, *Msh2* acts within MSNs as a genetic enhancer both of somatic *HTT* CAG expansions and of *HTT* CAG-dependent phenotypes in mice. These data suggest that the selective vulnerability of MSNs may be at least in part contributed by the propensity for somatic expansion in these neurons, and imply that intervening in the expansion process is likely to have therapeutic benefit.

## Introduction

Huntington’s disease (HD) is a dominantly inherited neurodegenerative disorder characterized by motor, cognitive and psychiatric symptoms [1]. The underlying cause is the expansion >35 repeats of a polymorphic CAG repeat within *HTT* gene that lengthens a glutamine tract in the huntingtin protein [2]. Stringent statistical analyses in a large HD patient data set indicate that the CAG expansion determines onset age in a fully dominant fashion with no evidence for a major role of either the wild-type allele or a second mutant allele [3]. While mutant huntingtin exerts its toxic effects in many brain regions as well as peripheral tissues over the course of the disease, medium-spiny GABA-ergic projection neurons (MSNs) in the striatum are the most vulnerable [4]–[6]. Therefore, the factors that contribute to this neuronal susceptibility are likely to provide clues to pathogenesis. Despite being caused by a single gene defect the disease is clearly complex, with a multitude of cellular pathways disrupted in response to mutant huntingtin [7]. Discerning those events that are critical to pathogenesis in order to design rational therapeutics remains a challenge.

An alternative to targeting downstream pathways that are disrupted during the course of disease is to target the CAG repeat mutation itself. Given that onset age and disease severity are highly correlated with the length of the expanded CAG repeat [3], [8], one would predict that reducing CAG length, even within the disease range, would have a beneficial effect. Notably, the mutant *HTT* CAG repeat exhibits both intergenerational and somatic instability [8]–[17]. The latter is highly biased towards expansions and is tissue-specific, with the greatest expansions seen in the striatum [13]. The striatum appears to be particularly susceptible to expansion in several trinucleotide repeat diseases [18]–[20], consistent with findings that expansion reflects an intrinsic property of this tissue rather than being a consequence of ongoing pathogenesis [21]. However, the further expansion of the mutant *HTT* CAG repeat in the striatum as well in other tissues susceptible to the effects of mutant huntingtin, is predicted to contribute to the pathogenic process. Indeed, longer somatic expansions in HD postmortem brain correlate with an earlier age of disease onset [17]. Therefore, the factors that modify repeat instability are predicted to modify disease and may lead to novel therapeutic targets.

To study the mechanisms underlying *HTT* CAG instability we have developed a series of *HTT* homologue (*Htt* or *Hdh*) CAG knock-in mice that replicate the genetic mutation in HD patients [22], [23]. Notably, these mice exhibit CAG length- and age-dependent, tissue-specific somatic expansion, with amongst the highest levels of instability seen in the striatum [21], [23], [24]. We have previously shown that striatal instability in *HdhQ111* mice is critically dependent on mismatch repair genes *Msh2* and *Msh3* [25], [26]. Significantly, constitutional knockout of either of these two genes delayed an early, dominant, CAG length-dependent phenotype in the striatum, the nuclear localization/epitope accessibility of mutant huntingtin, supporting the hypothesis that somatic expansions in target tissues contributes to the pathogenic process [25], [26].

Here, we sought to explore further the role of *Msh2* as a genetic modifier of *HTT* CAG repeat instability and pathogenesis. Given the particular susceptibility of MSNs to the disease process we have used a conditional knockout strategy to specifically delete the *Msh2* gene in this neuronal subtype of *HdhQ111* mice. This neuronal subtype-specific deletion of *Msh2*, allowed us to ask the following questions: 1. Is Msh2 required in MSNs to mediate *HTT* CAG expansion? 2. Is *Msh2* required in MSNs as a modifier of CAG repeat length-dependent mutant huntingtin localization and intranuclear inclusion phenotypes?

## Results

### Conditional Deletion of *Msh2* in Medium-spiny Striatal Neurons

**Figure 1:**
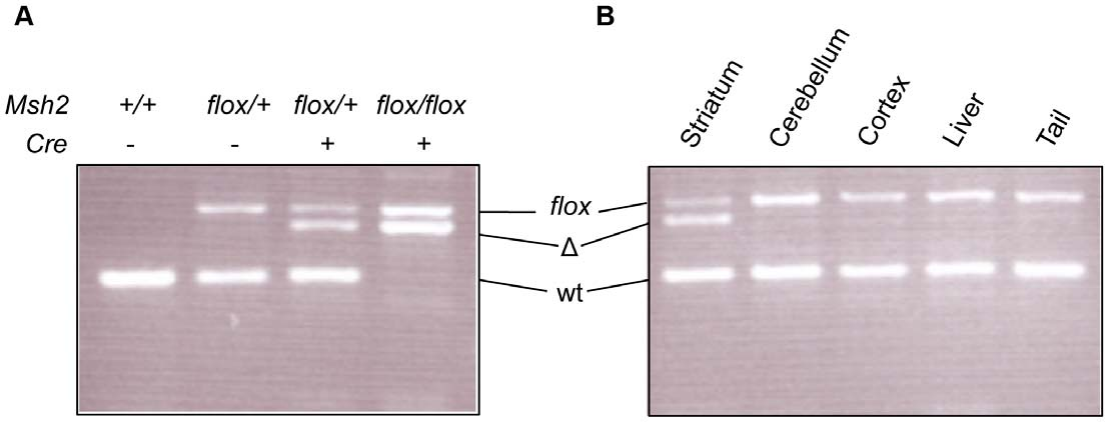
Conditional deletion of the *floxed Msh2* allele in the striatum. A. Genotyping for the conditional *Msh2* allele in genomic DNA extracted from striatum of *Msh2*+/+, *Msh2flox*/+, *Msh2flox*/+ *D9-Cre* and *Msh2flox*/flox *D9-Cre* mice. The deleted (Δ) *Msh2* allele is present only in mice harboring both the *Msh2flox* allele and the *D9-Cre* transgene. **B.** Genotyping for the conditional *Msh2* allele in genomic DNA extracted from five different tissues from a *Msh2flox*/+ *D9-Cre* mouse shows that the deletion is specific for the striatum. Mice were six weeks of age. *flox*: *Msh2* allele flanked by *loxP* sites; Δ:deleted *Msh2* allele; wt: wild-type *Msh2* allele.

To delete the *Msh2* gene we used a conditional *Msh2* knockout mouse line in which exon 12 that encodes part of Msh2’s essential ATPase domain is flanked by *loxP* sites (*Msh2flox*) [27]. To achieve specific deletion in MSNs we used a transgenic line (*D9-Cre*) in which Cre recombinase is expressed under the control of regulatory elements of the mouse *Ppp1r1b* gene encoding DARPP-32 [28]. Within the striatum, *D9-Cre* mice have been shown to express Cre specifically in MSNs from 5–6 weeks of age [28]. Crossing the *Msh2flox* and *D9-Cre* mice together demonstrated deletion of exon 12 of the *Msh2* gene in striatal DNA only in *Msh2flox* mice that also harbored the *D9-Cre* transgene (Figure 1A). Note that the undeleted (*floxed*) allele is still present in *D9-Cre* mice, which likely reflects contributions both from non-MSNs (interneurons and glia) that do not express the *Cre* transgene, and a small number of MSNs in which Cre-mediated recombination does not occur [28]. Comparison of genomic DNA isolated from different tissues (striatum, cerebellum, cortex, liver, tail) from *Msh2flox/+ D9*-*Cre* mice showed that deletion of *Msh2* exon 12 was specific to the striatum (Figure 1B). The absence of Cre-mediated recombination in other tissues is consistent with the very low number of cortical neurons and cerebellar purkinje cells in which Cre activity was previously observed and the general absence of recombination in peripheral tissues [28]. We will refer henceforth to the conditionally MSN-deleted allele, present in *Msh2flox D9-Cre* mice as *Msh2Δ.*

### Msh2 Protein is Eliminated in Medium-spiny Striatal Neurons

To examine the effect of MSN-specific deletion of *Msh2* on phenotypes elicited by an expanded *HTT* CAG repeat we established crosses with *HdhQ111* mice, *Msh2flox* mice, *D9-Cre* mice and *Msh2* null mice (Figure S1) to generate *HdhQ111*/+ mice with the following five genotypes: *Msh2*+/+, *Msh2*+/−, *Msh2Δ*/*Δ*, *Msh2Δ*/− and *Msh2*−/−, allowing us to compare directly the effect of MSN-specific deletion of *Msh2* and constitutional *Msh2* deletion. Note that while 50% of *Msh2*−/− mice die by 6 months of age due to tumor burden (largely in lymphatic tissue and the gut) [29] we did not observe any reduced viability or brain tumors in *Msh2Δ*/*Δ* or *Msh2Δ*/− mice that were aged as far as 10 months.

**Figure 2:**
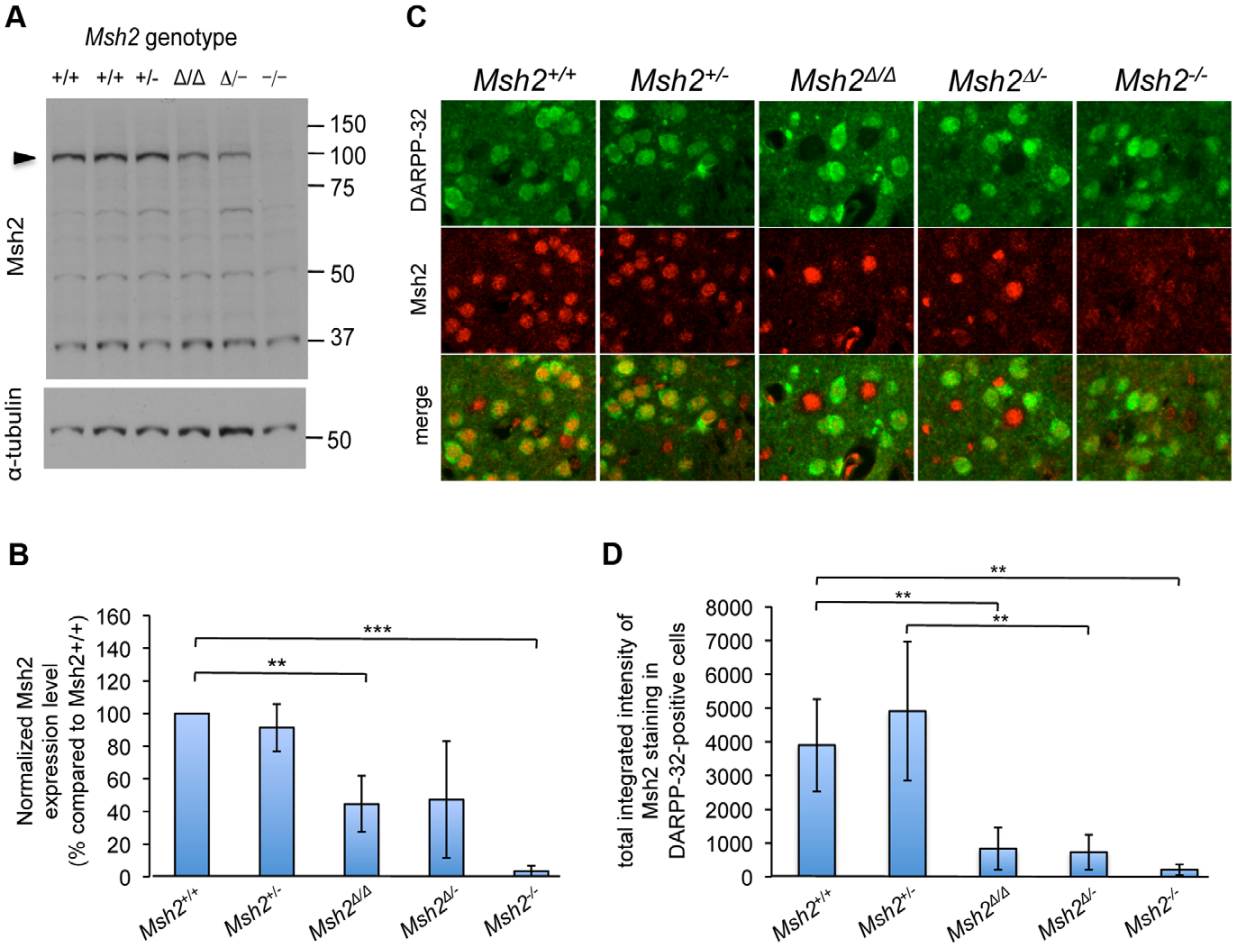
Msh2 protein levels in the striata of *Msh2* conditional knockout mice. A. Representative immunoblot of striatal lysates from five-month *HdhQ111*/+ mice with *Msh2*+/+, *Msh2*+/−, *Msh2Δ*/*Δ*, *Msh2Δ*/− and *Msh2*−/− genotypes probed with an anti-Msh2 antibody (upper panel). The membrane was stripped and re-probed with an anti-α-tubulin antibody (lower panel). Arrowhead indicates Msh2 protein. **B.** Quantification of immunoblots. The density of Msh2 bands from four immunoblots, each containing striatal protein lysates from different *HdhQ111*/+ mice with *Msh2*+/+ (n = 6, CAG 114, 116, 120, 123, 123, 124), *Msh2*+/− (n = 5, CAG 110, 114, 117, 119, 122), *Msh2Δ*/*Δ* (n = 3, CAG 118, 124, 126), *Msh2Δ*/− (n = 7, CAG 111, 113, 119, 120, 123, 124, 124), and *Msh2*−/− (n = 2, CAG 125 and a *Hdh*+/+ mouse) genotypes was quantified with QuantityOne software and normalized by the density of the corresponding α-tubulin bands. Four protein blots were run; on each, the Msh2/α-tubulin ratio was normalized to that of *Msh2*+/+ (100%) on that gel. Normalized Msh2/α-tubulin ratios were averaged across the 4 gels. Bars represent mean ±SD. **p<0.01; ***p<0.001. **C.** Fluorescent micrographs of striatal sections from five-month *HdhQ111*/+ mice with *Msh2*+/+, *Msh2*+/−, *Msh2Δ*/*Δ*, *Msh2Δ*/− and *Msh2*−/− genotypes co-stained with anti-MSH2 ab70270 and anti-DARPP-32 D32-6a antibodies. Note the significant overlap in DARPP-32 and Msh2 signals in *Msh2*+/+ and *Msh2*+/− striata, the loss of specific Msh2 signal in all cells in *Msh2*−/− striata and the specific loss of Msh2 signal in DARPP-32-positive cells in *Msh2Δ*/*Δ* and *Msh2Δ*/− striata. **D.** Mean integrated intensity of Msh2 immunostaining in DARPP-32-positive cells (total integrated intensity of ab70270 staining in D32-6a-positive cells normalized by the number of D32-6a-positive cells) in the striatum of the following five-month mice: *Msh2*+/+ (n = 6, CAG 113, 118, 119, 121, 123, 125), *Msh2*+/− (n = 3, CAG 114, 114, 123), *Msh2Δ*/*Δ* (n = 4, CAG 121, 121, 126, 129), *Msh2Δ*/− (n = 7, CAG 113, 121, 121, 122, 125, 125, 133), and *Msh2−/−* (n = 3, CAG 112, 120, 123). Bars represent mean ±S.D. *** p<0.0001, ** p<0.01, * p<0.05 (Student’s t-test).

We first determined the expression levels of Msh2 protein in striatal extracts from mice with *Msh2*+/+, *Msh2*+/−, *Msh2Δ*/*Δ*, *Msh2Δ*/− and *Msh2*−/− genotypes. Msh2 protein levels were reduced by ∼60% in *Msh2Δ*/*Δ* and *Msh2Δ*/− striatal extracts compared those in *Msh2*+/+ and *Msh2*+/− striatal extracts (Figure 2A,B). To assess more precisely the level of knockdown of Msh2 in MSNs we co-immunostained striatal sections with an antibody against DARPP-32 that specifically labels MSNs and an antibody against Msh2. This revealed the specific loss of Msh2 in MSNs in *Msh2Δ*/*Δ* and *Msh2Δ*/− mice (Figure 2C). Quantification of the immunofluorescent images (Figure 2D) showed significantly reduced levels of Msh2 in DARPP-32-positive MSNs in *Msh2Δ*/*Δ* compared to *Msh2*+/+ mice (p<0.01) and in *Msh2Δ*/− compared to *Msh2*+/− mice (p<0.01). There was no significant difference in levels of Msh2 in MSNs between *Msh2Δ*/*Δ* or *Msh2Δ*/− and *Msh2*−/− mice, indicating the complete loss of Msh2 in *Msh2Δ*/*Δ* and *Msh2Δ*/− MSNs. Note that the Msh2 protein level in *Msh2*+/− striata (Figure 2A, B) or MSNs (Figure 2C, D) is greater than 50% of that in *Msh2*+/+ striata, suggesting possible compensatory mechanisms, at least in striatal tissues/cells, by which the cell is able to regulate Msh2 levels to some degree.

### Msh2 Acts in Medium-spiny Striatal Neurons to Effect *HTT* CAG Expansion

**Figure 3:**
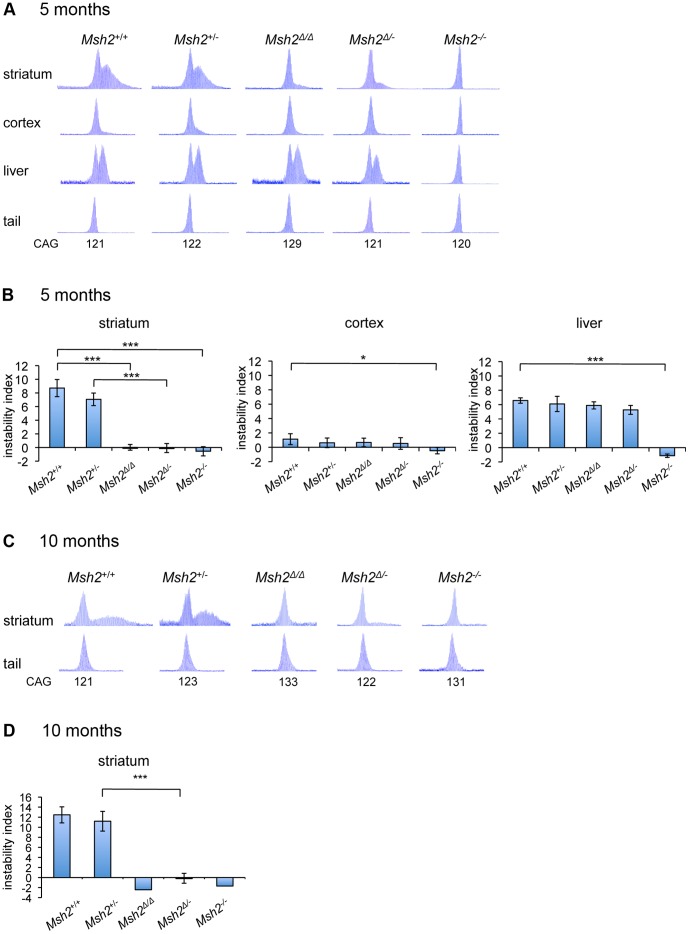
Deletion of *Msh2* in medium-spiny striatal neurons eliminates the majority of striatal *HTT* CAG expansions. GeneMapper traces of PCR-amplified *HTT* CAG repeats from striatum, cortex, liver and tail DNA of representative five-month *HdhQ111*/+ mice (**A**) or from striatum and tail of representative ten month *HdhQ111*/+ mice (**C**) with *Msh2*+/+, *Msh2*+/−, *Msh2Δ*/*Δ*, *Msh2Δ*/− and *Msh2*−/− genotypes. Constitutive CAG repeat lengths, as determined in tail DNA, are indicated. Instability indices were quantified from GeneMapper traces of PCR-amplified *HTT* CAG repeats from five-month striatum, cortex and liver (**B**) and ten-month striatum (**D**) of *HdhQ111*/+ mice with *Msh2*+/+, *Msh2*+/−, *Msh2Δ*/*Δ*, *Msh2Δ*/− and *Msh2*−/−genotypes. Five-month mice: *Msh2*+/+ (n = 6, CAG 113, 118, 119, 121, 123, 125), *Msh2*+/− (n = 4, CAG 114, 114, 120, 123), *Msh2Δ*/*Δ*(n = 5, CAG 113, 121, 121, 126, 129), *Msh2Δ*/−(n = 7, CAG 113, 121, 121, 122, 125, 125, 133) and *Msh2*−/− (n = 3, CAG 112, 120, 123). Ten-month mice: *Msh2*+/+ (n = 6, CAG 118, 121, 121, 123, 126, 134), *Msh2*+/− (n = 4, CAG 116, 118, 123, 131), *Msh2Δ*/*Δ* (n = 1, CAG 133), *Msh2Δ*/− (n = 7, CAG 115, 115, 117, 120, 121, 122, 123) and *Msh2*−/− (n = 1, CAG 132). Bars represent mean ± S.D. *** p<0.0001, * p<0.05 (Student’s t-test).

To investigate the effect of MSN-specific *Msh2* deletion on the somatic instability of the *HTT* CAG repeat *HdhQ111*/+ mice with *Msh2*+/+, *Msh2*+/−, *Msh2Δ*/*Δ*, *Msh2Δ*/− and *Msh2*−/− genotypes were aged to five months of age, a time-point at which we have previously observed significant accumulation of somatic expansions in *HdhQ111*/+ striata. GeneMapper traces obtained from PCR-amplification of the *HTT* CAG repeat (Figure 3A) showed a bimodal distribution of CAG repeat lengths in striatal DNA from both *Msh2*+/+ and *Msh2*+/− mice, as previously observed [26]. Also consistent with our previous studies of *Msh2* null *HdhQ111* mice [25], these somatic expansions were completely eliminated in striatal DNA from *Msh2*−/− mice. In striatal DNA from both *Msh2Δ*/− and *Msh2Δ*/*Δ* mice the somatically expanded repeats were dramatically reduced, although not eliminated as in *Msh2*−/− mice. Quantification of the GeneMapper traces (Figure 3B) revealed a significant reduction in instability in striata from *Msh2Δ*/*Δ* compared to *Msh2*+/+ mice (p<0.0001) and in striata from *Msh2Δ*/− compared to *Msh2+/−* mice (p<0.0001). As assayed using our quantification method, there were no significant differences in striatal instability between *Msh2Δ*/*Δ* and *Msh2*−/− mice (p = 0.18) or between *Msh2Δ*/− and *Msh2*−/− mice (p = 0.33). Consistent with the tissue-specific *Msh2* deletion shown in Figure 1B, *Msh2Δ*/*Δ* and *Msh2Δ*/− mice did not show any alteration in instability in liver or cortex, although instability in these tissues (high in liver and moderate in cortex) was eliminated in *Msh2*−/− mice.

To determine whether elimination of Msh2 in MSNs was sufficient to suppress somatic expansion over a more extensive time-period, we examined instability in *HdhQ111*/+ *Msh2Δ*/*Δ* and *HdhQ111*/+ *Msh2Δ*/− mice at ten months of age. While ten-month *Msh2*+/+ and *Msh2*+/− striata (Figure 3C, D) showed increased levels of instability compared to those at five months of age (Figure 3A, B), no obvious instability was apparent in either *Msh2Δ*/*Δ* or *Msh2Δ*/− striata at ten months (Figure 3C). Quantification (Figure 3D) revealed a significant difference in instability between *Msh2*+/− and *Msh2Δ*/− striata (p<0.0001). As there was only a single *Msh2Δ*/*Δ* at this age we were not able to perform any statistical analyses and it is formally possible that *Msh2Δ*/*Δ* mice may exhibit a wide variation in phenotype that is not apparent from the analysis of a single mouse (see also subsequent Results section). However, the essentially identical findings for this mouse compared to the seven *Msh2Δ*/− mice at the same age supports the conclusion that loss of *Msh2* in MSNs reduces instability. It is likely that at ten months of age the signal from the residual small population of unstable CAG repeats in *Msh2Δ*/*Δ* and *Msh2Δ*/− striata is too diffuse to be readily discernible and quantifiable by our method. Thus, it appears that while the residual unstable molecules likely continue to expand, the molecules stable at five months of age retain their stability at ten months of age in *Msh2Δ*/*Δ* and *Msh2Δ*/− striata. These data indicate that the majority of the *HTT* CAG striatal expansions occur in MSNs and that Msh2 expression within these neurons is critical to the expansion process over a period of at least ten months.

### Msh2 Acts in Medium-spiny Striatal Neurons as an Enhancer of the *HTT* CAG Repeat Length-Dependent Phenotypes

Previous data in HD patients and *HdhQ111* mice are consistent with the hypothesis that somatic expansions accelerate the *HTT* CAG-dependent pathogenic process [17], [25]. We have identified two CAG repeat-length dependent phenotypes in knock-in mice that would be predicted to be altered as a result of the loss of somatically expanded *HTT* CAG repeats; early (∼2.5 months) diffusely-immunostaining nuclear mutant huntingtin using the anti-huntingtin antibody EM48 and later (6–12 months) intranuclear inclusions of mutant huntingtin amino-terminal fragments [30]–[32]. While the direct consequences to the cell of either of these phenotypes are unclear, the observation that they are dominant, CAG repeat length-dependent and occur with a strong selectivity towards MSNs [30], [31] indicates that their underlying mechanisms are likely to be relevant to the pathogenic process in HD. Given the critical role of *Msh2* in mediating somatic expansion in MSNs we have tested whether *Msh2* is also a modifier of these two CAG length-dependent mutant huntingtin phenotypes. We previously showed that constitutional loss of Msh2 slowed the diffuse nuclear huntingtin phenotype in the striatum [25]. We have now developed a modified, quantitative assay to measure the time-dependent increase in diffuse nuclear mutant huntingtin in *HdhQ111*/+ mice using the anti-huntingtin monoclonal antibody mAb5374 (Figure S2). We have used this assay to determine the role played by MSN-expressed Msh2 in determining the diffuse nuclear mutant huntingtin phenotype. In our previous study [25] we did not analyze the effect of the *Msh2* null mutation on nuclear inclusions in *HdhQ111* mice as the reduced lifespan of the *Msh2* null mice precluded our ability to analyze this phenotype that was not apparent until ∼12 months on the genetic background of the original *HdhQ111* x *Msh2* null cross (mixed CD1/129Ola/FVB). Here, afforded by the earlier appearance of intranuclear inclusions on C57BL/6 backgrounds [32] we have also asked whether MSN-expressed Msh2 is a modifier of intranuclear inclusions.

**Figure 4:**
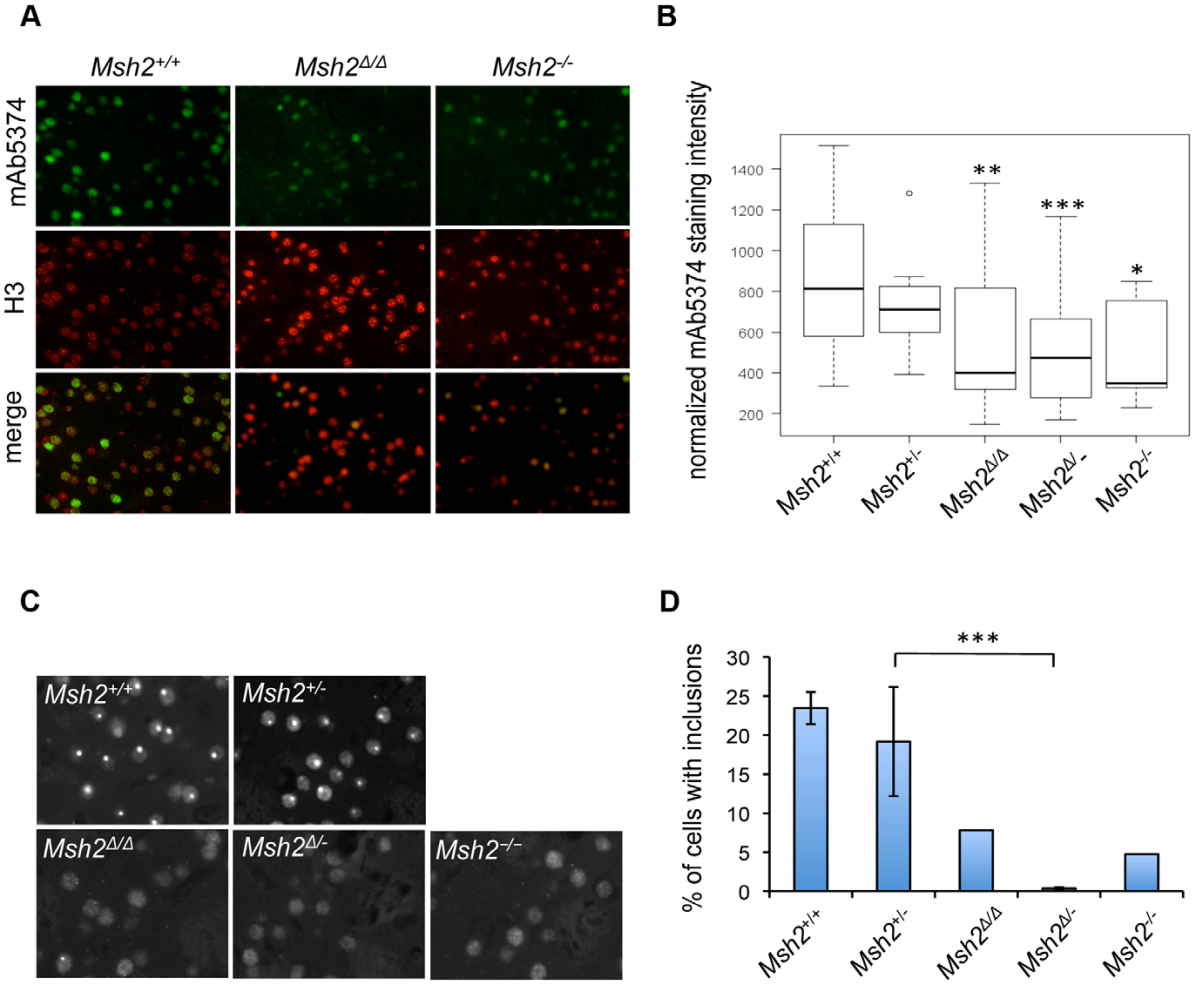
Deletion of Msh2 in medium-spiny neurons delays nuclear huntingtin phenotypes. A, B. Nuclear mutant huntingtin immunostaining is decreased in the striata of five-month old *HdhQ111*/+ mice with deletion of *Msh2* in MSNs. **A.** Fluorescent micrographs of striata double-stained with anti-huntingtin mAb5374 and anti-histone H3 antibodies for three CAG repeat length-matched mice (*Msh2*+/+ CAG 113, *Msh2Δ*/*Δ* CAG 112, *Msh2−/−* CAG 113). **B.** Box plot showing upper and lower quartiles, median and range for the normalized mAb5374 immunostaining intensity (total mAb5374 staining intensity normalized to the number of H3-positive nuclei). Outlier (circle) is defined by a standard interquartile method and is included in the analysis. Multiple regression analysis was used to determine the effect of *Msh2* genotype on mAb5374 staining using normalized mAb5374 intensity (continuous variable) as a dependent variable and *Msh2* genotype (discrete variable), constitutive CAG length (continuous variable) and position (medial versus lateral, discrete variable) as independent variables. Both constitutive CAG length (P<0.05) and medial versus lateral position (P<0.001) were significantly associated with normalized mAb5374 intensity. Asterisks above the bars indicate a significant difference from *Msh2*+/+ at a p-value cut-off of p<0.05(*), p<0.01 (**), p<0.001 (***) in the regression analysis. *Msh2Δ*/− was not significantly different from *Msh2*+/− (p = 0.18). The five-month mice used in the quantitative analysis are as follows: *Msh2*+/+ (n = 6, CAG 113, 118, 119, 121, 123, 125), *Msh2*+/− (n = 4, CAG 114, 114, 120, 123), *Msh2Δ*/*Δ* (n = 5, CAG 113, 121, 121, 126, 129), *Msh2Δ*/− (n = 7, CAG 113, 121, 121, 122, 125, 125, 133) and *Msh2*−/− (n = 3, CAG 112, 120, 123). Note that the relatively “weak” effect of the *Msh2*−/− genotype likely reflects the small number of mice of this genotype and hence the least accurate estimate of the relationship of mAb5374 intensity to CAG length in the regression analysis. **C, D.** Intranuclear inclusions are decreased in the striata of ten-month old *HdhQ111*/+ mice with deletion of *Msh2* in MSNs. **C.** Fluorescent micrographs of striata stained with mAb5374 from mice with *Msh2*+/+ (CAG 121), *Msh2*+/− (CAG 123), *Msh2Δ*/*Δ* (CAG 133), *Msh2Δ*/− (CAG 123) and *Msh2*−/− (CAG 132) genotypes. **D.** Quantification of the percentage of cells containing an inclusion (more than one inclusion per cell was rarely observed). The total number of cells was determined by co-staining with histone H3 (not shown). The ten-month mice used in the quantitative analysis are as follows: *Msh2*+/+ (n = 6, CAG 118, 121, 121, 123, 126, 134), *Msh2*+/− (n = 4, CAG 116, 118, 123, 131), *Msh2Δ*/*Δ* (n = 1, CAG 133), *Msh2Δ*/− (n = 7, CAG 115, 115, 117, 120, 121, 122, 123) and *Msh2*−/− (n = 1, CAG 132). Bars represent mean ±S.D.

Striatal sections from the same five-month and ten-month mice in which we determined somatic instability (Figure 3) were immunostained with mAb5374 (Figure 4). mAb5374 staining in five-month mice was found to depend both on the constitutive CAG repeat length as well as the position (medial versus lateral) within the striatum, with longer CAG lengths and lateral location resulting in increased staining (p<0.05 and p<0.001, respectively). Therefore, to determine the effect of *Msh2* genotype on mAb5374 staining we performed a multiple regression analysis, controlling for both constitutive CAG length and lateral versus medial position in the striatum. We found that, compared to *Msh2*+/+ mice, mAb5374 staining was reduced in both *Msh2Δ*/*Δ* (p<0.01) and *Msh2Δ*/− (p<0.001) mice as well as in *Msh2*−/− mice as predicted (p<0.05) (Figure 4A, B), although the difference in staining intensities in *Msh2*+/− and *Msh2Δ*/− striata did not reach statistical significance (p = 0.18).

In ten-month striata we observed a dramatic reduction in the number of mAb5374-positive intranuclear inclusions in *Msh2Δ*/*Δ*, *Msh2Δ*/− and *Msh2*−/− mice (Figure 4C, D). We were not able to perform statistical analyses of inclusion number in *Msh2Δ*/*Δ* or *Msh2*−/− mice due to an N = 1 for each of these genotypes, however, quantitative comparison of inclusion number in *Msh2Δ*/− versus *Msh2*+/− striata showed a highly statistically significant difference (p<0.001). Together, these data indicate that *Msh2* is a critical modifier that acts within MSNs to accelerate CAG repeat length-dependent mutant huntingtin phenotypes that are reflective of an ongoing HD-relevant pathogenic process.

## Discussion

We have demonstrated that *Msh2* acts in MSNs as a genetic enhancer of both *HTT* CAG repeat expansion and of *HTT* CAG repeat length-dependent mutant huntingtin phenotypes that occur as a part of an ongoing pathogenic process. As discussed previously [25], *Msh2* could, in principle, modify the disease process either indirectly via its role in modulating CAG repeat length, or directly, by some other unknown mechanism. Our findings indicate that *Msh2* does not accelerate the pathogenic process via detrimental effects to supportive glial cells or via detrimental systemic effects at the level of the whole organism, but rather acts in a cell-autonomous manner to influence phenotypes in MSNs. These data, therefore, are consistent with an indirect role for *Msh2* as a modifier of the pathogenic process via its effect on the somatic expansion of the *HTT* CAG repeat that occurs predominantly in MSNs. Our data support the hypothesis that somatic *HTT* CAG expansion in cells susceptible to the effects of mutant huntingtin accelerates the disease process, and further indicate that preventing somatic expansion in MSNs would have therapeutic benefit.

Laser capture microdissection studies in HD postmortem cortex and striatum have demonstrated that *HTT* CAG expansions occur both in neurons and glia, with neurons tending to have the longest expansions and greatest repeat length heterogeneity [14], [16]. Analysis of *HTT* CAG repeat length in microdissected neuronal and glial cells from striata of R6/2 *HTT* exon 1 transgenic mice supports the association of the most highly expanded repeats with neuronal cells [16]. Finer microdissection of neuronal populations within the striatum of knock-in mouse models of HD (*Hdh6/Q72* and *Hdh4/Q80)* demonstrated greater *HTT* CAG repeat expansion in a pan-neuronal (NeuN-positive) population compared to a nitric oxide synthase (NOS)-positive subpopulation of interneuron’s [14]. While the NeuN-positive cells served as a good approximation for MSNs that comprise >90% of striatal neurons these studies did not unequivocally demonstrate the occurrence of somatic expansions in MSNs. The present study supports the above data, and for the first time demonstrates that the bulk of the somatic expansions that occur in striatum arises in MSNs, and further demonstrates that these expansions are dependent on Msh2 expression within these neurons. Additional studies would be needed to determine whether the small proportion of expanded alleles that are present in the striata of the conditional knockout mice (Figure 3) are present in non-MSNs (neurons or glia) or in MSNs in which the *floxed Msh2* allele has failed to recombine. Regardless, it is clear that at a relatively early time-point (five to ten months of age), MSNs are the major cell type in the striatum exhibiting somatic expansions, while over time, it appears that expansions accumulate in other neurons and in glia [14], [16]. The finding that the earliest somatic expansions occur in the neuronal cell type that is selectively vulnerable in the disease is significant, supporting the proposition [33] that hyper-expansion of the *HTT* CAG repeat in MSNs contributes to their selective demise, and further suggesting that in *HTT* CAG mutation carriers, this is a process that may begin within months of birth.

Why are MSNs particularly vulnerable to repeat expansion? Several lines of evidence strongly indicate that instability in these neurons does not arise downstream of the pathogenic process: firstly, the spinocerebellar ataxia type 1 (*SCA1*) and type 3 (*SCA3*) CAG repeats [19], [20] and the myotonic dystrophy type 1 (*DMPK*) CTG repeat [18] show high levels of striatal instability despite the striatum being largely unaffected in these diseases; secondly, accelerating the pathogenic process in *HdhQ111* mice does not increase striatal *HTT* CAG instability [21]; thirdly, bioinformatic approaches based on correlating instability with global gene expression signatures predict the presence of a cellular environment permissive for repeat instability that is intrinsic to wild-type striata [21]. Although acute overexpression of polyglutamine-containing proteins has been found to influence repeat instability in fly models [34], the overwhelming evidence in accurate genetic HD disease models expressing mutant huntingtin at physiologically relevant levels is that factors other than the pathogenic process itself are primarily responsible for the somatic expansions that occur in MSNs.

Obvious factors that might underlie this cell-type specificity are Msh2 itself and its binding partner Msh3 that is also critical for striatal *HTT* CAG expansion [25], [26], [35], [36]. However, neither Msh2 nor Msh3 mRNA or protein levels showed any clear correlation with *HTT* CAG instability across tissues that vary in their degree of overall instability [21], although higher Msh3 protein in striatal neurons versus glia has been suggested [16]. Further investigation of the expression level, sub-cellular localization and activities of these proteins in MSNs versus striatal interneurons and glia is warranted. The stoichiometry of base excision repair proteins differs in striatum and cerebellum [37] and may contribute to the sensitivity of MSNs to somatic *HTT* CAG expansion. Interestingly, Xpa, a protein critical for transcription-coupled nucleotide excision repair (TC-NER), is required for instability of the *SCA1* CAG repeat in brain, but not in peripheral tissues [38]. It would be of interest to determine whether Xpa and TC-NER might play a role in contributing to the MSN-selective instability of the *HTT* CAG repeat. Transcription has also been shown to contribute to CAG/CTG instability [39]–[42]. Although steady state levels of *HTT* sense or antisense transcript do not clearly correlate with levels of tissue instability [43], it is possible that rates of sense, antisense or convergent transcription could play a role in determining the cell-type specificity of somatic instability in HD. Unbiased genome-wide analyses of factors associated with somatic instability, however, suggest that a combination of many factors is likely to contribute to the propensity of a particular tissue or cell-type towards somatic expansion [21].

In summary, the susceptibility of MSNs to both *HTT* CAG instability and HD pathogenesis strongly indicates that somatic expansion is relevant to the disease process in these neurons. Further experiments are required to unequivocally establish a role for somatic expansion as a disease modifier, to assess the spectrum of disease phenotypes that are subject to modification by somatic expansion as well as the extent to which they are modified. The prediction is that phenotypes that are CAG repeat length-dependent would be accelerated to some degree by further somatic expansion. Ongoing experiments are aimed at identifying CAG repeat length dependent phenotypes that will provide additional logical endpoints with which to test the hypothesis that somatic expansion accelerates the pathogenic process in mice.

How might somatic expansion be prevented in patients? In general, eliminating MMR proteins as a therapeutic strategy is undesirable due to the potential for tumor development, although targeting to the brain would likely reduce the tumor potential as cell-types in the periphery are the most susceptible to the somatic loss of MMR proteins. However, understanding the mechanism by which MMR proteins result in CAG expansion may provide a means to specifically intervene in this process without interfering in the role of these proteins in global genome maintenance. Alternative approaches could include the direct targeting of nucleic acid metabolic intermediates that occur during the expansion process, as recently indicated for the *DMPK* CTG repeat [44]. Finally, identification of additional factors that underlie *HTT* CAG instability in MSNs is an important goal as this would likely lead to novel targets for reducing somatic expansion in this vulnerable cell population.

## Materials and Methods

### Mice

This study was carried out in accordance with the recommendations in the Guide for the Care and Use of Laboratory Animals of the National Institutes of Health under an approved protocol of the Massachusetts General Hospital Subcommittee on Research Animal Care (protocol number 2009N000216). *HdhQ111* knock-in mice used in this study were on a C57BL/6J background [24] and were maintained by breeding heterozygous males to C57BL/6J wild-type mice from The Jackson Laboratories. The actual repeat size of the mice used in this study ranged from 110 to 134 CAGs. All analyses were performed on heterozygous *HdhQ111*/+ mice. *D9-Cre* transgenic mice contain a genomic fragment, comprising ∼2 kb of 5′ regulatory sequence, the endogenous ATG, and the introns and exons of the mouse *Ppp1r1b* gene encoding DARPP-32, driving the expression of Cre recombinase [28]. These mice were on a C57BL/6J background and were maintained as homozygotes. *Msh2flox* mice [27] that were used in this study had been backcrossed for nine generations to C57BL/6J. Constitutional *Msh2* null mice (*Msh2*−/−) [45] were on a C57BL/6N background. Note that we have not observed differences in *HdhQ111* instability or mAb5374 immunostaining phenotypes on C57BL/6J and C57BL/6N background (not shown), therefore the mixed C57BL/6J and C57BL/6N background is unlikely to be a confounding factor in our interpretation of the results.

To generate the mice of the appropriate genotypes for this study, *HdhQ111*/+, *Msh2flox*/+, *Msh2* null and *D9-Cre* mice were crossed together to obtain *Msh2*+/+ mice with two functional *Msh2* alleles, *Msh2*+/− mice with a single functional *Msh2* allele, *Msh2Δ*/*Δ* mice in which both alleles are deleted in MSNs, *Msh2Δ*/− mice with one constitutionally deleted *Msh2* allele and one *Msh2* allele deleted in MSNs, and *Msh2−/−* mice with two constitutionally deleted *Msh2* alleles. All of the mice analyzed for instability and mutant huntingtin phenotypes with these genotypes were *HdhQ111/+* heterozygotes. A detailed schematic of the crosses involved is shown in Figure S1.

### Genotyping and Analysis of Somatic Instability

Genomic DNA was isolated from tail biopsies at weaning for routine genotyping analysis or from adult tissues (either fresh or fixed) for somatic instability analysis, using the PureGene DNA isolation kit (Gentra, Minneapolis, MN, USA). Genotyping of the *HdhQ111* knock-in allele was carried out as described previously [26]. *Msh2flox* and *D9-Cre* mice were genotyped as described [27], [28]. *Msh2* null mice [45] were genotyped in a three-primer PCR assay using 1 µM primer MSH2A.

(5′ CCCTCCTGTTGAGCCATCTTA), 0.75 µM of primer MSH2B (5′ GCCAGCTCATTCCTCCACTC) and 0.5 µM of primer MSH2C (5′ TTCGCTGCTTGTCTCTGGAAT), 200 µM dNTPs, 1 x Qiagen PCR buffer with 1.5 mM MgCl_2_ and 0.6 units of *Taq* polymerase (Qiagen). Cycling conditions were 95°C for 9 mins; 40 cycles of 94°C for 45 sec, 56°C for 45 sec, 72°C for 45 sec; 72°C for 5 mins, generating a 188 bp product from the wild-type allele (MSHA/MSHC primers) and a 300 bp product from the *Msh2* null allele (MSH2B/MSH2C primers). *HdhQ111* CAG repeat size was determined using human *HTT* CAG-specific primers as previously described [26] with the forward primer fluorescently labeled with 6-FAM (Perkin Elmer). Products were resolved using the AB13730xl automated DNA analyzer (Applied Biosystems). GeneMapper v3.7 with GeneScan 500-LIZ as internal size standard was used to assign repeat size, defined as the highest peak in the GeneMapper trace. All runs included the same control DNAs of known *HTT* CAG repeat size. Somatic instability was quantified from the GeneMapper traces as described previously [21]. Briefly, GeneMapper peaks <10% of the height of the highest peak were excluded. For peaks exceeding the 10% background threshold, normalized peak heights were calculated by dividing the peak height by the sum of all peak heights over background. The change in CAG length of each peak from the highest peak in tail DNA (main allele) was determined, the normalized peak height was multiplied by the CAG change from the main allele, and these values were summed to generate an instability index that represents the mean CAG repeat length change in the population of cells being analyzed.

### Immunohistochemistry

Primary antibodies were: mouse monoclonal anti-huntingtin mAb5374 (Chemicon), and rabbit polyclonal anti-histone H3 (Abcam ab1791), rabbit polyclonal anti-MSH2 (Abcam ab70270) and mouse monoclonal anti-DARPP-32 (D32-6a; a kind gift from Dr. Angus Nairn). Immunostaining was performed on 7 mm coronal sections of periodate-lysine-paraformaldehyde (PLP)-perfused and -post-fixed, paraffin-embedded brains. Perfusion and tissue processing/embedding methods have been described previously [30]. One hemisphere of the brain was embedded for sectioning and the other was used for dissection of striatum, cortex and cerebellum for analysis of somatic instability. Sections chosen for immunostaining were aligned with respect to their anterior/posterior location in the brain. The sections were deparaffinized, rehydrated and subjected to heat-mediated epitope retrieval (Na-citrate buffer pH 6.0) followed by quenching of endogenous peroxidase with 0.3% H_2_O_2_/methanol for 30 min at room temperature and blocked in 3% normal horse serum (NHS) in TBS for 1h at room temperature.

For detection of diffusely immunostaining nuclear huntingtin, incubation with mAb5374 (1∶100 in 1% NHS/TBS) was carried out overnight at 4°C. mAb5374 signal was then amplified using the TSA Biotin System (Perkin Elmer) according to manufacturer’s instructions. Briefly, sections were incubated sequentially with biotinylated anti-mouse IgG (Vectastain Elite ABC kit; Vector Laboratories) at 1∶200 dilution in 1% NHS/TBS for 1 h at RT, with Streptavidin-conjugated horseradish peroxidase (Streptavidin-HRP, TSA Biotin System, Perkin Elmer) at 1∶100 in 1% NHS/TBS for 30 min at RT, and with biotinylated Tyramide Amplification reagent (TSA Biotin System, Perkin Elmer) at 1∶75 in the diluent provided for 20 min at RT. mAb5374 staining was detected by Streptavidin-Alexa Fluor 488 (Invitrogen) at 1∶500 in 1% NHS/TBS for 1 h at RT. For double staining with mAb5374/anti-histone H3 antibody, sections were incubated with both primary antibodies overnight at 4°C, and histone H3 staining was detected with donkey anti-rabbit Alexa Fluor 546-conjugated secondary antibody (Invitrogen, 1∶1000) added together with Streptavidin-Alexa Fluor 488. For detection of huntingtin inclusions, sections were incubated with mAb5374 (1∶200 in 1% NHS/TBS) overnight at 4°C. Signal was detected using goat anti-mouse-HRP (TSA kit #2, Invitrogen) at 1∶50 in 1% NHS/TBS for 1 h at RT, followed by Alexa Fluor 488-conjugated Tyramide (TSA kit #2, Invitrogen) at 1∶100 in the diluent provided with the TSA kit #2, for 25 min at RT. Detailed conditions were according to manufacturer’s instructions (TSA kit #2, Invitrogen). For double staining with mAb5374/anti-histone H3 antibody, sections were incubated with both primary antibodies overnight at 4°C, and histone H3 staining was detected with donkey anti-rabbit Alexa Fluor 546-conjugated secondary antibody (Invitrogen, 1∶1000) added after Tyramide-Alexa Fluor 488. For double staining with anti-DARPP-32/anti-Msh2 antibodies, sections were incubated with both primary antibodies (D32-6a at 1∶200 and ab70270 at 1∶500 in 1%NHS/TBS) overnight at 4°C, followed by sequential amplification of Msh2 and DARPP-32 signal. First, Msh2 signal was amplified using the TSA Biotin System, and quenching of HRP activity (0.1% sodium azide/0.3% H_2_O_2_ in TBS for 30 min at RT) was performed before the final addition of streptavidin-Alexa Fluor 555. Then, DARPP-32 signal was amplified by incubating with goat anti-mouse-HRP (TSA kit #2, Invitrogen) at 1∶100 and Tyramide-Alexa Fluor-488 (TSA kit #2, Invitrogen) at 1∶100 in the diluent provided with the TSA kit #2, for 25 min at RT. Detailed conditions were according to manufacturer’s instructions (TSA kit #2, Invitrogen). ‘No primary antibody’ and ‘single primary antibody’ control experiments demonstrated the specificity of the Alexa Fluor-555 and Alexa Fluor-488 signals for Msh2 and DARPP-32, respectively.

Sections were mounted in ProLong Gold antifade reagent (Invitrogen). Fluorescent microscopy was performed with a Zeiss Axioskop 2 microscope equipped with AxioCamMRm camera and AxioVision 4.6 image acquisition software, using Plan Apochromat 20x/0.8 M27 or Plan Neofluar 40x/0.75 Ph2 objectives. Images that were to be quantified and compared were taken with the same exposure times.

### Quantification of Immunohistochemical Data

Cell Profiler 2.0 r10997 cell image analysis software was used for quantification of mAb5374 and Msh2 immunostaining intensity. Four micrographs were taken from each mouse (one from medial and one from lateral striatum in two consecutive sections located on the same slide), each micrograph representing an area of 220×170 µm. Total (integrated) intensity of mAb5374 staining was measured in all mAb5374-positive nuclei in each area and normalized by the total number of nuclei (as determined by the number of all histone H3-positive nuclei) in the same area. The resulting value, representing the mean intensity of mAb5374 staining per nucleus, was averaged for each mouse (for medial and lateral striatal areas separately as well as for all four striatal areas). Note that there was some variation in histone H3 intensity both within and between mice (see for example Figure 4). However, this did not vary systematically with genotype, nor did it correlate with the number of histone H3-positive nuclei. We normalized against the number of histone H3-postive nuclei in order to control for potential differences in cell density in different coronal sections, However, the number of histone H3-positive nuclei between sections analyzed did not vary considerably, indicating that our sections were reasonably well matched in terms of their anterior/posterior positions. For Msh2 staining, total (integrated) intensity of Msh2 staining was measured in DARPP-32-positive cells in each area and normalized by the number of DARPP-32-positive cells. The resulting value, representing the intensity of Msh2 staining per MSN, was averaged for each mouse (from the four striatal areas as described above). The number of nuclear huntingtin inclusions in the striatum of 10 month old mice was quantified in fluorescent micrographs of mAb5374/anti-H3-stained striatal sections (four 435×435 µm striatal areas per mouse, as described above) using ImageJ software and normalized to the number of H3-positive cells.

### Immunoblot Blot Analysis

Striata were homogenized in RIPA buffer supplemented with 5 mM EDTA and protease inhibitors (Halt protease Inhibitor Cocktail, ThermoScientific) by mechanical grinding with disposable pestle (Fisher Scientific) and further sonication (Branson sonifier, power level 3.5, two 10-sec pulses on ice). The homogenates were kept on ice for 30 min and then clarified by centrifugation for 30 min at 14000 rpm. Protein concentrations were measures using Bio-Rad DC protein assay kit. Striatal extracts (30 ug per lane) were resolved by SDS-PAGE in Novex 10% Bis-Tris gels (Innvitrogen). Msh2 protein was detected by immunoblotting using anti-MSH2 antibody ab70270 (Abcam) at 1∶4000 in 5% non-fat milk/TBS/0.1% Tween-20. Alpha-tubulin, as detected by 1∶5000 DM1A (Cell Signaling Technology) in 5% milk/TBS/0.1% Tween-20, was used as loading control. Quantification was done by measuring the density of Msh2 and α-tubulin bands with QuantityOne software with subtraction of local background.

### Statistical Analyses

Pairwise comparisons of instability indices, intranuclear inclusions and Msh2 protein levels were determined using a Student’s t-test (Excel). In these comparisons, *Msh2Δ*/*Δ* mice were generally compared to *Msh2*+/+ mice, and *Msh2Δ*/− mice were compared to *Msh2*+/− mice as this latter comparison provides a more stringent test of the effect of the conditional deletion on a background of a single constitutionally deleted *Msh2* allele. To control for the effects of constitutive CAG length and striatal position (medial versus lateral) in the determination of the effect of *Msh2* genotype on mAb5374 immunostaining intensity multiple regression analysis was carried out using normalized mAb5374 intensity as the dependent (continuous) variable and *Msh2* genotype (discrete variable), constitutive CAG length (continuous variable) and position (discrete variable) as independent variables using the R statistical package (version 2.7.2).

## Supporting Information

Figure S1 **Breeding Scheme.** The breeding scheme shows how we generated *HdhQ111*/+ mice of the five *Msh2* genotypes (*Msh2*+/+, *Msh2+/−, Msh2Δ/Δ*, *Msh2Δ/−*, *Msh2*−/−) that were used in this study using the four transgenic/knock-in alleles (*HdhQ111*, *Msh2* null, *Msh2flox*, *D9-Cre*). Breeders in the various crosses are shaded in green and separated by a green “X”. The genotypes of the resultant mice from these crosses that were analyzed are indicated in black type. Shown in red type/boxes are the five *Msh2* genotypes using the final terminology we describe throughout the manuscript. Note that mice with a single functional *Msh2* allele could be generated in a number of ways and were pooled as heterozygotes (*Msh2*+/− mice in red type). Similarly, mice with two functional *Msh2* alleles could be generated in more than one way and were pooled as wild-types (*Msh2*+/+ mice in red type).
(TIF)

Figure S2 **Quantitative fluorescent assay to detect the time-dependent increase in mAb5374-positive nuclear mutant huntingtin immunostaining in the striata of** ***HdhQ111/+*** **mice (C57BL/6J).** Left: Fluorescent micrographs of striata of three *Hdh+/+* mice (upper panel) and three CAG repeat length-matched *HdhQ111/+* mice (lower panel), double-stained with anti-huntingtin mAb5374 and anti-histone H3 antibodies. CAG repeat numbers: 121 (2.3 months), 120 (3.3 months), 121 (5.0 months). Note that this antibody does not stain huntingtin in wild-type (*Hdh*+/+) mice. Right: Quantification of the mAb5374 nuclear immunostain in *HdhQ111*/+ striata, normalized to the number of H3-positive nuclei. 2.3 mo N = 4; 3.3 mo. N = 3; 5.0 mo N = 3. Bars are mean±S.D.
(DOCX)
