# Supplementary figures and images for: Msh2 Acts in Medium-Spiny Striatal Neurons as an Enhancer of CAG Instability and Mutant Huntingtin Phenotypes in Huntington’s Disease Knock-In Mice
Source: PLoS One. 2012 Sep 7;7(9):e44273. doi: 10.1371/journal.pone.0044273 (PMC3436885; doi:10.1371/journal.pone.0044273)

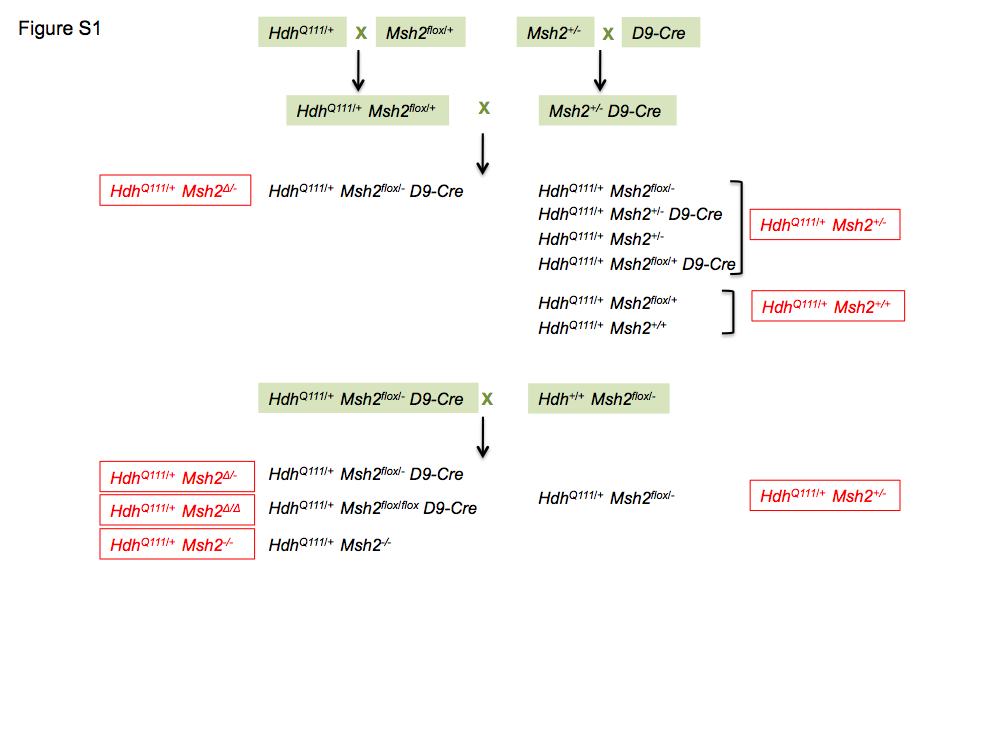

Supplement: Figure S1 — Breeding Scheme. The breeding scheme shows how we generated HdhQ111/+ mice of the five Msh2 genotypes (Msh2+/+, Msh2+/−, Msh2Δ/Δ, Msh2Δ/−, Msh2−/−) that were used in this study using the four transgenic/knock-in alleles (HdhQ111, Msh2 null, Msh2flox, D9-Cre). Breeders in the various crosses are shaded in green and separated by a green “X”. The genotypes of the resultant mice from these crosses that were analyzed are indicated in black type. Shown in red type/boxes are the five Msh2 genotypes using the final terminology we describe throughout the manuscript. Note that mice with a single functional Msh2 allele could be generated in a number of ways and were pooled as heterozygotes (Msh2+/− mice in red type). Similarly, mice with two functional Msh2 alleles could be generated in more than one way and were pooled as wild-types (Msh2+/+ mice in red type). (TIF) [file pone.0044273.s001.tif]

**Figure S2**

**
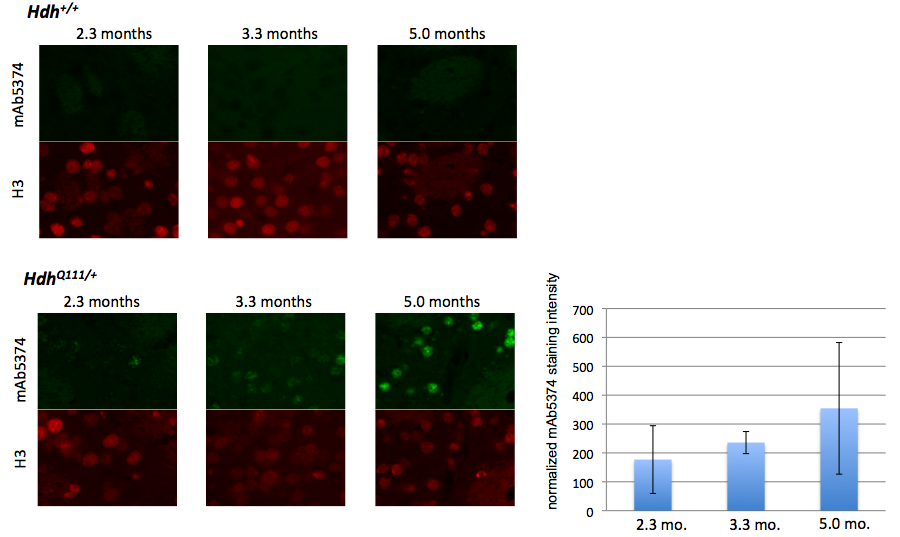
**

Supplement: Figure S2 — Quantitative fluorescent assay to detect the time-dependent increase in mAb5374-positive nuclear mutant huntingtin immunostaining in the striata of HdhQ111/+ mice (C57BL/6J). Left: Fluorescent micrographs of striata of three Hdh+/+ mice (upper panel) and three CAG repeat length-matched HdhQ111/+ mice (lower panel), double-stained with anti-huntingtin mAb5374 and anti-histone H3 antibodies. CAG repeat numbers: 121 (2.3 months), 120 (3.3 months), 121 (5.0 months). Note that this antibody does not stain huntingtin in wild-type (Hdh+/+) mice. Right: Quantification of the mAb5374 nuclear immunostain in HdhQ111/+ striata, normalized to the number of H3-positive nuclei. 2.3 mo N = 4; 3.3 mo. N = 3; 5.0 mo N = 3. Bars are mean±S.D. (DOCX) [file pone.0044273.s002.docx]
